# Transdisciplinary breastfeeding support: Creating program and policy synergy across the reproductive continuum

**DOI:** 10.1186/1746-4358-3-16

**Published:** 2008-08-04

**Authors:** Miriam H Labbok

**Affiliations:** 1 Center for Infant and Young Child Feeding and Care, Department of Maternal and Child Health, University of North Carolina, School of Public Health, Chapel Hill, North Carolina, USA

## Abstract

This paper was presented at the symposium on Breastfeeding and Feminism: A Focus on Reproductive Health, Rights and Justice. It underscores the power and potential of synergy between and among organizations and individuals supporting breastfeeding, the mother-child dyad, and reproductive health to increase sustainable breastfeeding support. These concepts were brought together to lay the groundwork for working group discussions of synergy in program and policy actions.

## The issue

Reproduction may be defined as the successful creation of the next generation of individuals of the same species who are also able to reproduce. Given the importance of breastfeeding for ensuring the health and survival of the next generation, its impact on fertility, and its impact on maternal health, it is arguable that breastfeeding is as much a part of the reproductive continuum as are conception, pregnancy, birth and family planning. Many talented individuals from women's collectives, health systems, and policy-making bodies are doing excellent work to support and protect women at various points on the reproductive continuum. However, when any one point on the continuum is addressed, without consideration of the other parts of the continuum, the action cannot yield the most sustainable and effective result. And yet, despite the many commonalities in the philosophies, policies and programs that address maternal survival, birthing practices, family planning, or breastfeeding, there has not as yet been a concerted effort to ensure synergy. Sustainable and ever growing action in these arenas may depend on identification of synergies and building on mutual strengths.

This article explores reproductive health, rights and justice as an intergenerational continuum, more than as a women's issue or a child issue, alone. As a group of doctors and lawyers, lactation consultants, government health workers, public health specialists and community activists, feminists, scholars, mothers, fathers and children, let us consider together the possibility that each of us might peer outside of the "silos" of our own interest areas to create new and increased cross-sectoral synergy in program and policy.

While there is a general familiarity with terms such as cross-disciplinary and multi-disciplinary, the concept of transdisciplinarity is relatively new. An understanding of the derivation of this term is helpful in understanding its importance for work on reproduction and breastfeeding.

• **Disciplinary: **Of, or relating to, a specific field of academic study.

• **Multidisciplinary: **Of, relating to, or making use of several disciplines at once.

• **Interdisciplinary: **Of, relating to, or involving two or more academic disciplines that are usually considered distinct.

• **Cross-Disciplinary: **Integrative learning.

It is important to note that these terms do not necessarily involve initial or ongoing working together throughout all stages of a process. When multiple disciplines do not work together from the start, it can lead to unexpected conflicts and occasionally quite negative outcomes. For example, many disciplines are addressing the international HIV/AIDS issue today, included the disease-specific AIDS community, the Gender Awareness community, and child survival community.

• The goals of HIV/AIDS disciples include ensuring that every possible case of HIV/AIDS is prevented or treated, and that those living with HIV/AIDS are given all the support that they need. In attempting to prevent every chance of mother-to-child transmission by whatever means possible, and, this group has worked to eliminate breastfeeding by all HIV-positive women.

• The goals of gender awareness disciples include ensuring that gender issues are considered in all programming, and that rights of all genders are respected. For example, "Prevention of Mother to Child Transmission (PMTCT)" terminology is considered gender insensitive, "blaming the mother." Repeated requests to change the term to "pediatric HIV/AIDS" or even "vertical transmission" have gone unheeded. Another activity considered to be gender insensitive was the decision to concentrate on testing only pregnant women, rather than all women, sending a clear message that infants' health is prioritized above women's health.

• The goals of child survival disciples include ensuring the reduction of preventable morbidity and mortality, and increasing rates of survival, growth and development. From this perspective, the goal is HIV-free survival, and this is best served by promotion of exclusive breastfeeding and other proven child survival interventions for all, and consideration of HIV/AIDS secondarily as only one of the causes of mortality amongst many.

Bringing various disciplines together once they are entrenched in their own paradigms often may seem impossible, and later attempts to create synergy too frequently result in entrenchment and less than optimal policy and programme development.

## Suggested responsive actions

### Transdisciplinarity

Jean Piaget coined the word "transdisciplinarity" [1]. He called for reaching beyond interdisciplinary coordination, stating "concerning interdisciplinary discourse, we hope to see a higher level emerge, 'transdisciplinarity', which would not settle for interactions or reciprocities between specializations, but which would internalize such interaction within an overall construct, and break down the walls between disciplines." Transdisciplinarity guides problem perceptions and solutions. Alternatively stated, a transdisciplinary approach would require that many disciplines come together not only to address a predefined problem from each one's own perspective, but rather to together define the problem to be addressed.

If the HIV, gender awareness and child survival groups had taken a transdisciplinary approach initially, they may have found initial commonalities and decided on a different set of goals and messages, such as:

• Formula use expends dollars needed for treatment or prevention.

• Where it is not possible to formula feed safely, exclusive breastfeeding is the best option as it could cut transmission by half.

• Test and treat all women. This may result in decisions not to become pregnant, hence reducing the risk for the next generation, and would help the survival of all family members.

Transdisciplinarity enhances our competency to address and resolve controversial problems. A decision to employ a transdisciplinary approach might have avoided the programmatic conflicts that remain unresolved in the field. Whether the topic for discussion is HIV, gender or the risks posed by lack of breastfeeding, specialized scientific knowledge is always involved. This knowledge must, however, be contextualized so that it becomes part of problem resolution competencies and can be applied in their conflicts of interest and value. A transdisciplinary approach to messaging would have facilitated working together, and would have supported coordination and synergy of efforts, actions and programs that would have met needs across disciplines.

### Creating paradigm shifts towards mutual visions and synergy

**Figure 1:**
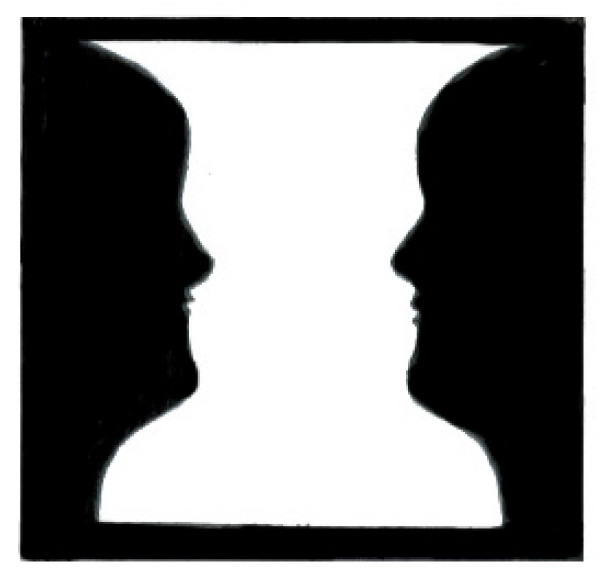
**Vase? Or two profiles?**. Our initial perspective may make it difficult to view things from a new perspective, but it is possible.

It is not easy to step outside of our individual disciplinary or special interest boxes. Altering perceptions is difficult, but it may be an essential step in creating collaborative approaches. Visual perception differences are a common part of psychology 101: in Figure 1, who sees a vase and who sees two people facing each other?

It is not always easy to visualize the image differently than it was originally perceived; but once you do see the alternate image, it becomes more difficult to return to your original perspective. These visual images ask the viewer for paradigm shift in how a single image is viewed. Is there any question, then, that we all have to struggle to see each other's viewpoint on complex issues? The analogy between viewpoints on such images, and viewpoints from disciplinary perspectives, is this: once one has seen things from a perspective different than one's own, it is harder to go back to one's original unilateral stance.

Today, I am asking each of you to come along with me to see if we each can make a paradigm shift on the issues in the reproductive continuum – family planning, pregnancy and birthing and breastfeeding. These are issues that are intimately, biologically, gender linked in women's lives, and yet ones that are generally divided up to be addressed by a variety of different professional disciplines. Despite the impact of child spacing on birthing success, of birthing practices on breastfeeding success, and of breastfeeding on child spacing, we are offered family planning services by a gynecologist, birth attendance by an obstetrician or midwife, and baby care by a pediatrician. Having these "silos" of care, each with its own paradigm and priorities, may lead to conflicting messages, and hence, may undermine the search for mutuality in goals, and collaboration.

Initiatives to support breastfeeding, such as the Baby-friendly Hospital Initiative [2] or initiatives to support mother-friendly maternity practices, such as Mother/Baby-friendly Initiative, or even mother support, must actively seek synergy for optimal impact [3]. In fact, the revised Baby-friendly Hospital Initiative includes standards and goals for birthing practices, for breastfeeding-friendly communities, and guidance for birth spacing, in addition to reconfirming the original Ten Steps to Successful Breastfeeding [4]. Breastfeeding policy and programs would benefit from common messaging and protocols for breastfeeding and mutual support, no matter which discipline is involved. However, this would demand an active decision on the part of healthcare workers and policy-makers to end the "silo" approach.

Existing paradigms can exert an even stronger polarization between family planning and breastfeeding interest groups. This divide is especially unfortunate given the inextricable biological relationships between the two practices. Breastfeeding is a proximate determinant of fertility, and allowing breastfeeding practices to deteriorate will increase fertility rates significantly [5]. One would think, therefore, that breastfeeding would always be a factor of great interest to family planning professionals, whether in program development, in research design, or in policy formation, and that family planning would be essential component for action by breastfeeding supporters in order to ensure an adequate duration of breastfeeding, without a mother suffering the nutrition and health taxing of a concurrent pregnancy. However, examples of active collaboration between family planning programs and breastfeeding support programs are, paradoxically, rare. There is, however, one example of active planning for synergy that has occurred: the research and development of the Lactational Amenorrhea Method (LAM) as an introductory method of family planning [6]. For women using LAM, breastfeeding is the physiological basis of a family planning method that indicates when another method must be introduced.

The development of LAM is a personal story. As a young physician working in the Population Office at USAID, having been mentored by Dr. Cicely Williams, and recognized by La Leche League International recognition for my work on breastfeeding, I had been sensitized both to the importance of breastfeeding and to the potential negative impact of estrogens on breastfeeding. One of my first projects was the development of a combined oral contraceptive (COC) program with the Ministry of Health in Morocco, a country where breastfeeding was the norm; USAID at the time advised introduction of COCs at two weeks postpartum, despite the evidence that this might disrupt breastfeeding. Today, the decision might be different; both family planning and breastfeeding researchers have identified the synergies of these practices in terms of health and spacing outcomes. However, at the time, the blinders were on, and the early introduction of COCs was initiated. This illustration of the need for out-of-the-box thinking inspired my interest in the development of a family planning method that would encourage synergy rather than competition between family planning and breastfeeding advocates.

**Figure 2:**
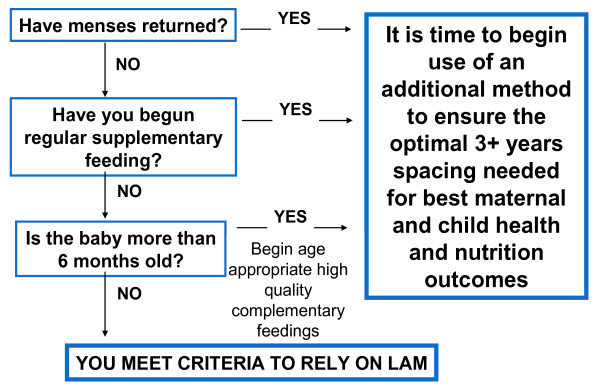
**Lactational amenorrhea method**. This method of family planning evolved from transdisciplinary thinking: How can we address the need to support breastfeeding and child spacing? (figure derived from references in [12]).

The development of LAM began when the same Office sponsored a research session to discuss the issue of the timing of contraception postpartum: if introduced too early during breastfeeding it would be duplicative and possibly detrimental, leading to too early cessation of family planning use, but introduction too late could predispose to an unhealthy, short birth interval. We started funding research on this issue, and I soon left USAID to continue research on this issue at Hopkins. By 1988, when Family Health International and Rockefeller sponsored the first consensus meeting on the issue [7], sufficient data were available from several research centers to identify the three criteria that were later codified as a method algorithm at a meeting held by the Institute for Reproductive Health at Georgetown [6] (see Figure 2).

Despite its proven efficacy and acceptability [8,9], LAM is not as yet always listed as a method in texts. Nor do many breastfeeding texts address the need for child spacing for optimal health outcomes. However, WHO's "Family Planning: A Global Handbook for Providers" includes LAM as a highly efficacious method, breastfeeding support groups often include LAM as another benefit of exclusive breastfeeding [10,11]; and USAID is renewing its dedication to this method given the increasing need to integrate and synergize family planning and child survival service delivery [12]. We are clearly beginning to see signs that synergy is possible and happening.

Two additional "silos" where collaboration, cooperation and synergy are beginning to emerge in support of breastfeeding are *Safe Motherhood and Newborn Initiative *and *Child Survival *strategies. Optimal birth intervals help to protect positive birth outcomes for mothers and babies, and improve the nutritional status and odds of survival in the short and long terms [13]. And clearly, whatever the child survival strategy employed, whether Accelerated Child Survival and Development, Integrated Management of Childhood Illnesses, or Expanded Programme on Immunization, child survival will benefit from synergy with the number one life-saver as per the Lancet Series on Child Survival: exclusive breastfeeding support [14].

### Policy areas upon which to build synergy and action

**Figure 3:**
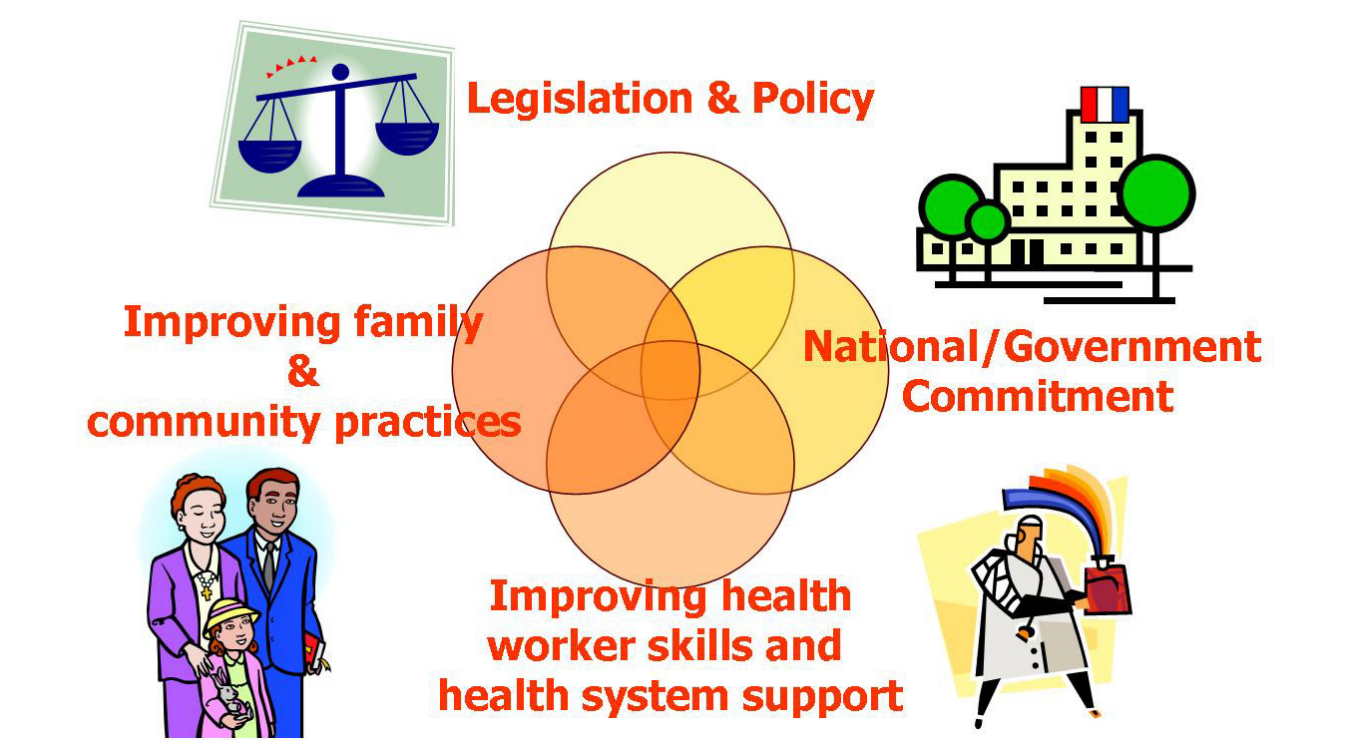
**The four pillars**. Frameworks for Strategic Planning where Breastfeeding and Family Planning and Reproductive Rights could synergize in support of program and policy: The 4 Action Areas ("pillars") for Synergy Consideration (figure derived from UNICEF and WHO materials).

There is no shortage of "jumping off points" for a coalition of individuals and organizations committed to healthier, happier mothers and babies. However, four policy "pillars" have been defined as a solid base for sustainable change. These pillars include: national/state government commitment, legislation and policy, health worker training and health system support, and family and community support (see Figure 3). As we examine a way forward in generating action ideas to build synergy, it may be useful to keep these in mind. Examples of policy actions in support of breastfeeding that demand coalitions of support include:

• National/governmental Commitment: This may be supported using women's and children's rights as arguments for change;

• Legislation/policy for maternity protection and paid leave, health insurance coverage, freedom to breastfeed as children need, and protection against aggressive advertising of infant formula. Coalitions including labor unions and business coalitions, such as the National Business Group for Health, could partner with breastfeeding and feminist groups to achieve these policy goals;

• Health training and services improvement necessitate cooperation and partnership among State Health Departments, health professional associations, accrediting organizations, and academic faculties to ensure that preventive medicine, breastfeeding, and attention to women's equity are included in undergraduate training for all health workers;

• Policy in support of family/community must include attention to social support for birth spacing and motherhood, as well as the sharing of social marketing and advocacy across sectors. Such policy dictating action would include building with existing socially-oriented NGOs, no matter what their primary social goal is.

Here in the US, in North Carolina, eight "Recommended Breastfeeding Action Areas," that address the four pillars have already been established in the NC Blueprint for the Protection, Promotion and Support of Breastfeeding [15]:

1. Encouraging the adoption of activities that create breastfeeding-friendly communities;

2. Creating a breastfeeding-friendly health care system;

3. Encouraging the adoption of breastfeeding-friendly workplaces;

4. Assisting childcare facilities in promoting, protecting, and supporting breastfeeding;

5. Advocating for insurance coverage by all third-party payers for breastfeeding care, services, and equipment when necessary;

6. Involving media and using social marketing and public education to promote breastfeeding;

7. Promoting and enforcing new and existing laws, policies and regulations that support and protect breastfeeding; and,

8. Encouraging research and evaluation on breastfeeding outcomes, trends, quality of care, and best practices.

Globally there are at least three policies that could serve as a foundation for planning activities that serve as a construct for synergy of breastfeeding and family planning. The first is the Millennium Development Goals for improving maternal and child health, including gender equity and reproductive justice as underlying needs [16]. The second is the Partnership for Safe Motherhood and Newborn Health [17]. The third policy is the Global Strategy for Infant and Young Child Feeding and the Innocenti Declarations, where advocates could ensure that sufficient attention is paid to family planning, birthing, and breastfeeding [18].

### Concepts for planning synergized action steps

The reason I have emphasized paradigms is that each of us must actively recognize our own paradigms – our entrenched viewpoints – then work to perceive the issue from a new stance. If you will recall the story of my experience in Morocco, the request to synergize family planning and breastfeeding would have necessitated a paradigm shift for all who were working on family planning or child survival. At that time, there was no perceived benefit in seeking synergy between these efforts. Today, with diminished resources for public health, being open to new approaches to creating the much-needed synergy among breastfeeding, family planning and feminism can only serve us well.

**Figure 4:**
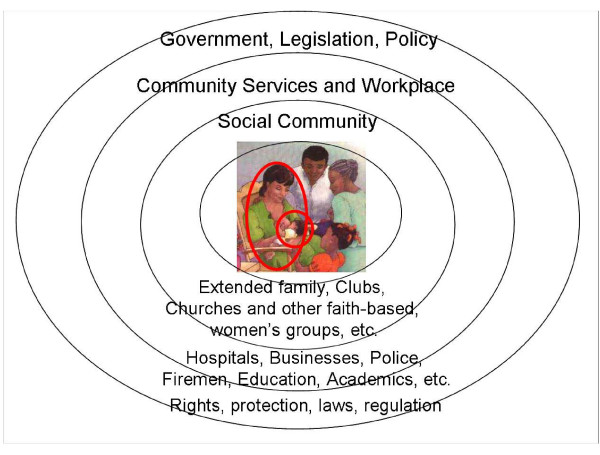
**The socio-ecologic framework**. Comprehensive, multi-level, multi-sectoral protection, promotion and support for breastfeeding. The socio-ecological model can be used to identify points of synergy and intervention; Construct of this model is similar to the rights-based model, both starting with consideration of the child and mother.

Two additional ways to more easily visualize points of potential synergies are through exploration of the continua, both social and chronological. The socio-ecological model is a visual description of the continua of interactions among the individual and the various strata of society: family, social community, workplace culture, and legislative and other government authorities. This model is useful in identifying interactions among various impacts on health, and in identifying loci for intervention (see Figure 4). The construct is conceptually similar to the rights-based model, which recognizes that support must be in place in each stratum in order to actualize the rights of the individual. It is possible to see how various programs may emanate from one stratum, and the logic of creation of synergy rather than conflicting impacts.

**Figure 5:**
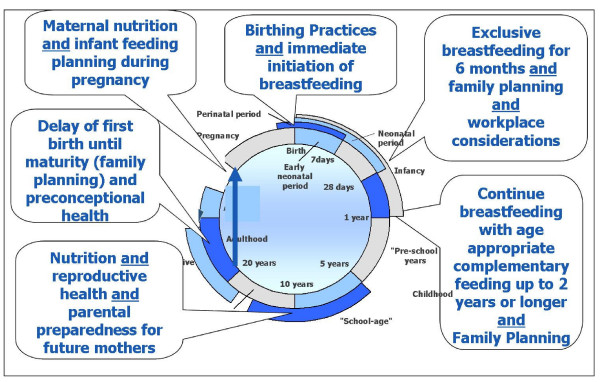
**The intergenerational life-cycle**. The intergenerational life-cycle may help us identify areas where program and policy interventions and be synergized, and rights and justice considered.

The chronological intergenerational life-cycle approach may also support the development of new integrated and synergized interventions to promote health, rights and justice by accepting that there are sequelae of programs and policies that reach beyond the moment, and are visited upon the future of individuals and into subsequent generations (see Figure 5). If we consider this, it is clear that no intervention can allow itself to stand alone, but rather there must be developed and implemented in cooperation among those who have contact at any point to ensure ongoing best and most sustainable outcomes.

## Conclusion

This presentation is intended to stimulate thinking about the difficulties in stepping outside of our primary areas of interest, and to offer frames of reference for considering where synergies might be identified and acted upon. Dedicated individuals and organizations who care about equity can find areas of synergy to enhance dynamic social action in support of reproductive health, rights and justice, regardless of their primary objectives. Specific actions, such as inclusion of LAM as an introductory method towards adequate child spacing in breastfeeding, family planning, and child survival programming, are encouraged.

Our mission here today is to support the reproductive continuum, including health, rights and justice and addressing birth, breastfeeding and family planning. The support of the reproductive continuum is, literally, a matter of life and death. The areas of synergy and action that emerge with transdisciplinary approaches may best support effective and sustainable action inhealth, rights and social justice for mothers, children, and perhaps, for the most naturally synergized unit – the mother/child dyad.

## Competing interests

The author declares that they have no competing interests.
